# Transdermal fentanyl for the treatment of pain caused by osteoarthritis of the knee or hip: an open, multicentre study

**DOI:** 10.1186/1471-2474-6-31

**Published:** 2005-06-15

**Authors:** Xavier Le Loët, Karel Pavelka, Ute Richarz

**Affiliations:** 1 Centre Hospitalier Universitaire de Rouen, Hôpitaux Rouen, Rouen, France; 2 Institute of Rheumatology, Prague, Czech Republic; 3 Janssen-Cilag Medical Affairs (Europe, Middle East & Asia), Baar, Switzerland

## Abstract

**Background:**

This study was designed to evaluate the utility of transdermal fentanyl (TDF, Durogesic^®^) for the treatment of pain due to osteoarthritis (OA) of the knee or hip, which was not adequately controlled by non-opioid analgesics or weak opioids. The second part of the trial, investigating TDF in patients with rheumatoid arthritis (RA) is reported separately.

**Methods:**

Current analgesia was optimised during a 1-week run-in. Patients then received 28 days treatment with TDF starting at 25 μg/hr, with the option to increase the dose until adequate pain control was achieved. Metoclopramide was taken during the first week and then as needed.

**Results:**

Of the 159 patients recruited, 75 with OA knee and 44 with OA hip completed the treatment phase, 30 knee and 18 hip patients entered the one-week taper-off phase. The most frequently used maximum dose of TDF was 25 μg/hr. The number of patients with adequate pain control increased during the run-in period from 4% to 27%, and further increased during TDF treatment to 88% on day 28. From baseline to endpoint, there were significant reductions in pain (p < 0.001) and improvements in functioning (p < 0.001) and physical (p < 0.001) and mental (p < 0.05) health. Scores for 'pain right now' decreased significantly within 24 hours of starting TDF treatment. TDF was assessed favourably and 84% of patients would recommend it for OA-related pain. Nausea and vomiting were the most common adverse events (reported by 32% and 26% of patients respectively), despite prophylaxis with metoclopramide, which showed limited efficacy in this setting.

**Conclusion:**

TDF significantly increased pain control, and improved functioning and quality of life. Metoclopramide appeared to be of limited value in preventing nausea and vomiting; more effective anti-emetic treatment may enable more people to benefit from strong opioids such as TDF. This study suggests that four weeks is a reasonable period to test the benefit of adding TDF to improve pain control in OA patients and that discontinuing therapy in cases of limited benefit creates no major obstacles.

## Background

Osteoarthritis (OA) is a slowly developing articular disease, characterized mainly by cartilage degeneration, which is reflected clinically by a gradual development of joint pain, stiffness, and loss of full range of movement. OA is the most common disease to affect synovial joints, being a major cause of musculoskeletal pain, reduced quality of life and disability. About 40–60% of patients with radiological osteoarthritic changes suffer from clinical symptoms of pain, stiffness and loss of mobility [1], and around 55% of patients with OA report pain as the worst aspect of the disease [2]. OA is strongly associated with ageing and, with an increasing elderly population, of major socioeconomic importance.

Current treatments for OA include a wide range of non-pharmacological, pharmacological and surgical options, although evidence to support their effectiveness is variable and there are no curative treatments. Therapies focus on reducing symptoms such as pain and stiffness, and minimalizing functional limitation and disability [3]. However, pain control is the primary aim of treating patients with OA and, in evaluating symptoms, pain should be the primary outcome variable [4]. Non-steroidal anti-inflammatory drugs play an important role in the pharmacological management of OA [5]. However, their lack of efficacy or potential toxicity may limit their use, in particular, the withdrawal of certain Cox-2 inhibitors has restricted the choice of therapies [3,5-7], and problems of persisting pain remain. Pain from OA may be caused by factors other than inflammation [8], therefore the logical next step in the treatment of OA-related pain is the use of strong opioids. Within a management programme aimed at improving physical and social function, guidelines recommend their use when other appropriate therapies have failed to provide adequate pain relief over a reasonable period of time [9-13].

Transdermal fentanyl (TDF), providing systemic delivery of fentanyl at a constant rate for 72 hours [14], has been shown to be effective in controlling pain and improving some quality of life parameters for people with chronic non-malignant pain [15-18]. The efficacy of opioids in controlling pain in patients with OA has been demonstrated in three randomized controlled trials [19-21]. Moreover, a prospective study to investigate the efficacy and tolerability of TDF in 243 patients with severe OA pain of the knee and/or hip demonstrated significant reductions in pain at rest and on movement, and provided evidence of functional improvement [22]. Very few patients needed doses higher than the 25 μg/hr starting dose after 30 days of treatment [22].

The present study was undertaken to evaluate the utility and safety of TDF for the treatment of pain associated with RA or with OA of the knee or hip, which was not adequately controlled by NSAIDs, Cox-2 inhibitors, paracetamol or weak opioids at optimal doses. As it was an open-label study, it was not designed to prove efficacy of the treatment but to investigate practical aspects of opioid therapy, such as the usefulness of a test-period or concomitant use of antiemetic therapy and to serve as a pilot for a double-blind trial in this patient population. A test period for evaluation of a patient's response to opioid treatment is recommended [10] and in this study lasted 4 weeks. Results from the total study population and from patients with RA will be reported elsewhere. This paper reports the effects of TDF on pain and functioning in patients with OA of the knee or hip.

## Methods

### Patient selection

All participants were outpatients requiring supplementary analgesia because of moderate or severe pain, which was not adequately controlled with paracetamol, NSAIDs, Cox-2 inhibitors or weak opioids (e.g. tramadol or codeine). Because non-opioid analgesia is not always taken at sufficient doses to achieve pain control [24] the trial employed a run-in period during which analgesia was optimised. Patients who still had moderate or severe pain at the end of this period could enter the main treatment phase of the study. Patients had to be over 50 years old, have OA of the knee or hip and meet the OA criteria of the ACR [23]. They had to have radiographic evidence of OA, and be waiting for hip or knee replacement as indicated by an orthopaedic surgeon. If participants were taking corticosteroids and/or NSAIDs they had to have received a stable dose for at least three months before screening and expect to remain on a stable dose for the duration of the trial.

Patients were excluded from the study if they had received regular treatment with a strong opioid (e.g. morphine) or had received more than the maximum recommended dose of weak opioids or other analgesics in the four weeks before the study. Strong opioids other than fentanyl, supplementary weak opioids or other treatments that might alter the degree or nature of pain could not be started during the study. Patients were excluded if they had continuous pain of non-arthritis origin, or had undergone surgery/arthroscopy within 3 months, intra/peri articular injections for arthritis pain (e.g. steroid injection) within 6 weeks, or arthrocentesis within 4 weeks of the study start.

### Study design

Screened patients satisfying the selection criteria each gave written informed consent before inclusion in this international, open, prospective trial. The study was carried out in accordance with the latest revision of the Declaration of Helsinki and Good Clinical Practice and was approved by independent local ethics committees.

During the one-week run-in period, non-opioid analgesic treatment was increased to the maximum tolerated or maximum recommended dose, while weak opioids were kept stable. All patients with pain control rated as poor or very poor on a 5-point scale at the end of the run-in period started treatment for 28 days with transdermal fentanyl (TDF, Durogesic^®^) at a dose of 25 μg/h. Patches were replaced every 72 hours (3 days). Previous non-opioid analgesia was continued and kept stable, but weak opioids were discontinued. The dose of TDF could be increased in steps of 25 μg/h every 72 hours (days 3,6 and 9) if required (no maximum dose specified) until adequate pain control was achieved. After 28 days or if necessary for other reasons, e.g. if side effects occurred or the treatment was not effective, a similar downward titration regimen was employed. Paracetamol 500 mg tablets were provided for supplementary analgesia and could be used in doses of up to 4 g/day. With the exception of paracetamol, other non-opioid analgesics were kept stable and no short acting opioids were added during down titration.

Metoclopramide 10 mg three times a day was given to all patients for the prevention of nausea and vomiting during the first week of treatment, after which it was taken as needed.

### Assessments

Patients were evaluated in the clinic at screening (day -7), at baseline (day 0), on days 7, 14 (optional because some clinicians see their patients only every 4 weeks) and on day 28 (trial end), on other days if dose adjustment was necessary, and at the end of a one or two week tapering-off period.

The primary efficacy variable was pain control, evaluated weekly on a five-point assessment scale ranging from very poor to excellent. For this assessment, the investigator presented the question "would you rate your pain control today as being excellent, good, moderate, poor or very poor?" Patients also completed a pain assessment questionnaire (shortened version of the Wisconsin Brief Pain Inventory (WBPI) [25]) (10-point rating scale: 0, best to 10, worst). The degree of pain after 24 hours of treatment was assessed by asking patients about the amount of pain that they had 'right now'. Patients also recorded pain intensity in a diary on a five-point scale.

Patients completed a treatment assessment questionnaire consisting of 10 items scored on a Likert scale. The acute version of the SF-36 quality of life questionnaire [26] including eight quality of life domains (physical functioning, physical role limitations, emotional role limitations, social functioning, body pain, general mental health, vitality and general health perceptions) was completed after the run-in phase and at day 28. Functionality of patients was assessed at the same time points using the Western Ontario and McMaster Universities Osteoarthritis Index LK 3.1 (WOMAC questionnaire) [27]. This has 24 questions that are each evaluated on a 5-point severity scale (0, none to 4, extreme), it assesses three areas: pain (5 questions), stiffness (2 questions) and functional impairment (17 questions). Maximum scores for each section differ and, therefore, scores are normalized by weighting severity to assist interpretation.

### Statistical analysis

There was no formal sample size calculation. A safety analysis was performed using data from all patients entering the trial. An intent-to-treat analysis, comprising all OA patients with at least one post-baseline measurement of the primary endpoint (pain control) and treated at least once with trial medication, was also performed [28].

The ANCOVA model was used to analyse the change from baseline to endpoint, and the influence of baseline values. The ANCOVA model was also used to determine differences by centre. The Wilcoxon signed-rank test was used to compare intragroup results and results at each time point or endpoint with baseline, where applicable. Statistical tests were interpreted at the 5% significance level (two-sided).

## Results

### Patient characteristics

A total of 159 patients, 102 with OA of the knee and 57 of the hip, were recruited into the study from 47 centres in 11 countries and started treatment with TDF. Patient characteristics are shown in Table 1. All but three patients used analgesic treatment in the month before screening and the treatments were similar for hip and knee groups. The most commonly used non-opioid analgesics were paracetamol 21%, diclofenac 20% and rofecoxib 10%. The most commonly used weak opioid was tramadol (33%). During this time, half the patients (50%) used a non-opioid only; 28% used a combination of a non-opioid and weak opioid and 18% a weak opioid only.

**Table 1:** Patient characteristics

	**OA knee**	**OA hip**
N (baseline)	102	57
ITT analysis population	91	52
Mean age (years) ± SE (range)	68 ± 0.9	66 ± 1.2
	(49–88)	(47–87)
Previous medication (% of patients):		
Non-opioids	81	79
Weak opioids	50	40

Concomitant medication with possible analgesic effects during the treatment phase included paracetamol (69%), NSAIDs (45%), weak opioids (33%), Cox-2 inhibitors (13%), other analgesics (8%), steroids (6%), immunomodulating drugs (1%), and strong opioids (1%). Of those taking weak opioids, most took them on the first day of treatment only. Only two patients used opioid rescue medication and had to be considered as protocol violators. There was a reduction in the use of NSAIDs (from 45% to 18%) and Cox-2 inhibitors (9%) and paracetamol (15%) after the first 24 hours of treatment. Rescue medication was required by 59% of participants, of whom almost all used non-opioids, particularly paracetamol. Most patients suffered from concomitant diseases: 59% of the patients had currently active cardiovascular diseases, 50% musculo-skeletal, 48% genito-urinary, 47% endocrine, and 19% gastrointestinal disease. The most frequently used non-analgesic concomitant therapies during treatment were metoclopramide 49% and omeprazole 12%.

### Discontinuations

**Figure 1:**
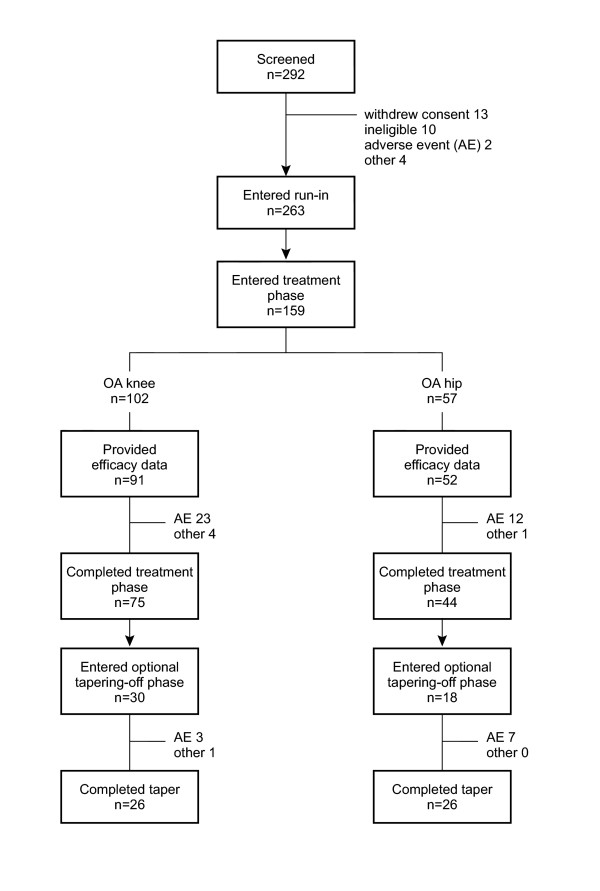
Patient disposition.

Of the 159 participants recruited, 25% withdrew from the trial during the treatment phase. Reasons for withdrawal were adverse events (35), insufficent response (1) and other reasons (4). Around half of the drop-outs (14%, 21 of the 35 due to adverse events) occurred in the first week of TDF treatment (Figure 1). Of the 48 patients who started the optional tapering-off phase, 11 (23%) discontinued prematurely (10 because of adverse events and one was lost to follow-up/surgery). Patients were treated for an average of 22.3 ± 0.92 days.

### Study medication

All patients started at a dose of 25 μg fentanyl/h and daily doses ranged from 25 to 125 μg/h. The maximum dose was used by only one patient. The mean daily dose for week 1 was 26 μg/h, which increased slightly to 37 μg/h in week 4. Over half (54%) of all patients used 25 μg/h as a maximum dose during the study (61% OA knee, 42% OA hip).

### Evaluation of efficacy

#### Primary efficacy variable

**Figure 2:**
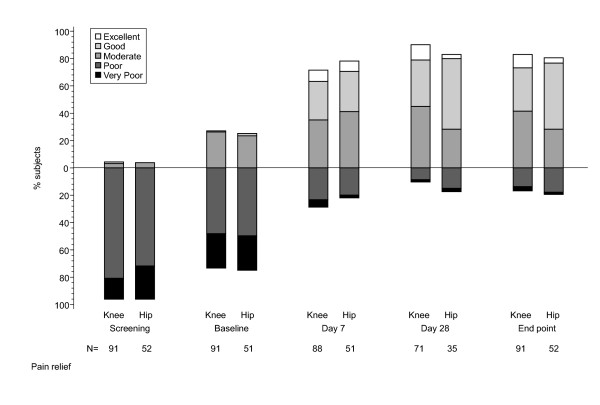
Pain control assessment.

Adequate pain control was defined as a score of 'moderate', 'good' or 'excellent' on the 5-point pain control assessment scale. The proportion of patients with adequate pain control increased during the one-week run-in period (during which doses of non-opioid analgesia were optimised) from 4% to 27%. These patients continued in the study and accounted for the majority of protocol violators. At baseline, 25% of patients reported very poor pain control, 48% poor and 25% moderate pain control, and there was no notable difference between those with OA knee and OA hip (Table 2). A further increase in the proportion of patients with adequate pain control was observed after TDF treatment, particularly in the first week of treatment (to 74%) when 37% patients reported moderate, 29% good, and 8% excellent pain control. Adequate pain control was reported by 80% and 88% patients on days 14 and 28, respectively. At endpoint, 83% of patients considered their pain controlled, with 37% reporting moderate, 38% good, and 8% excellent pain control (Table 2 & Figure 2). About 10% more patients with OA hip reported good or excellent pain control at endpoint than those with OA knee. However, of the patients who already experienced adequate pain control after the run-in phase (OA knee, 24 moderate and one good pain control; OA hip, 12 moderate and one good pain control), 50% of both groups improved further during TDF treatment. Overall, 81% of participants with OA hip and 75% with OA knee improved from baseline to endpoint by at least one pain category.

**Table 2:** Pain control assessments (number of patients and percentage in each category)

	**Screening **N (%)	**Baseline **N (%)	**Endpoint **N (%)
Very poor	27 (19)	36 (25)	4 (3)
Poor	110 (77)	68 (48)	21 (14)
Moderate	5 (3)	36 (25)	53 (37)
Good	1 (1)	2 (2)	54 (38)
Excellent	0	0	11 (8)

#### Effect size

An effect size was calculated by assigning numerical values to the pain categories (1 = very poor, 2 = poor, 3 = moderate, 4 = good, 5 = very good). This gives a mean pain control score at baseline of 2.03 (95% CI 1.9, 2.2), rising to 3.33 (3.2, 3.5) at endpoint and a mean change from baseline of 1.3 (SD ± 1.14). This gives an absolute effect size of 1.14.

#### Secondary efficacy variables

##### Pain control

Patients reported a significant reduction in pain from baseline to endpoint for each WBPI item at every time point (p < 0.001). The mean reduction in 'pain at its worst' was 1.8 points (from 8.1 to 6.3), 'pain at its least' was 1.6 points (from 4.4 to 2.8), and 'pain on average' was 2.0 points (from 6.4 to 4.4). The amount of trouble or bother the pain was causing also decreased by 2.7 points on average (from 7.2 to 4.5). The mean reduction in 'pain right now' was 2.6 points (from 6.1 to 3.5) from baseline to endpoint. A significant reduction in 'pain right now' was reported as early as 24 hours after baseline (1.3 points, from 6.0 to 4.7).

From patients' diaries, the mean score for degree of pain was significantly decreased at each time point, and from severe pain (score 3) to moderate pain (score 2) from the run-in period to endpoint (p < 0.001). Results were similar for the patients' highest score for their degree of pain. Thus, while at baseline 58% (79/137) reported severe/extreme pain, 4% (5) mild, and only two patients were without pain, by endpoint 41% (56/138) reported moderate pain, 30% (41) mild and 7% (9) no pain. There was little difference between the OA knee and OA hip group.

##### Treatment assessment

In their assessment of treatment (total n = 125, OA knee 82, OA hip 43), 63% of patients rated TDF positively with respect to pain control and 84% would recommend TDF for their type of pain. Most patients were satisfied with its convenience of use (93% thought it easy/extremely easy to use; 85% were very/somewhat pleased by the way it's used), and 53% considered side effects were not an issue. In general, there was a difference of less than 10% between the OA knee and OA hip groups. In assessing how they had felt over the past week, the percentage of all patients who answered good or very good increased during the study from 7% (10/142) during the run-in period to 32% (31/97) in week 4, and their scores at all time points were significantly better than before treatment (p < 0.001). By the end of the study, help with basic activities was required by only 28% of patients, with 49% relying less on their helper.

### Quality of life

For the 122 patients who completed the SF-36 quality of life questionnaire, there were statistically significant improvements in all domains from baseline to endpoint, including overall physical health (p < 0.001) and mental health (p < 0.05) (Table 3). Despite optimization of previous treatment, quality of life scores were low at baseline, with the patients' underlying osteoarthritis particularly affecting role physical and bodily pain. It was in these two areas that patients showed greatest absolute improvement with TDF treatment.

**Table 3:** Quality of life (SF-36 scores)

**Domain**	**n**	**Score at baseline **(Mean ± SE)	**Mean change between baseline and endpoint **(95% CI)	***p-*value**
*Physical functioning*				
Physical functioning	120	30.0 ± 2.15	4.0 (-0.09, 7.99)	<0.05
Role physical	119	11.8 ± 2.38	15.8 (8.89, 22.62)	<0.001
Bodily pain	122	23.7 ± 1.54	17.1 (13.21, 20.97)	<0.001
General health	119	44.1 ± 2.09	3.6 (0.09, 7.05)	<0.05
				
*Mental health*				
Vitality	119	34.6 ± 1.74	6.3 (3.24, 9.45)	<0.001
Social functioning	122	50.3 ± 2.55	7.9 92.99, 12.79(	<0.05
Role emotional	117	34.9 ± 3.97	10.8 (1.85, 19.81)	<0.05
Mental health	118	53.0 ± 2.06	4.7(1.28, 8.03)	<0.05
				
*Summary measures*				
Physical health	111	27.0 ± 0.69	4.1 (2.73, 5.54)	<0.001
Mental health	111	41.7 ± 1.17	2.5 (0.46, 4.64)	<0.05

### WOMAC

The mean score for all 24 questions from the three summary parameters (pain, stiffness and physical functioning) improved significantly from baseline to endpoint for all groups (total population p < 0.001, knee p < 0.001, and hip p < 0.05 for all questions) (Table 4). The percentage of patients in the combined group and in the knee sub-group who reported no pain, stiffness or physical difficulties increased for all items. A similar increase occurred in the OA hip group except for 'stiffness after first awakening', 'rising from bed' and 'getting in/out of the bath' which showed little change.

**Table 4:** WOMAC scores (mean normalised score ± SE, all changes from baseline are statistically significant, p < 0.001 in all cases)

	**Baseline**	**Endpoint**	**Change from baseline to endpoint **(95% CI)
Pain	6.3 ± 0.15	4.6 ± 0.21	-1.7 (-2.05, -1.26)
Stiffness	6.1 ± 0.19	4.7 ± 0.23	-1.4 (-1.89, -1.0)
Physical functioning	6.6 ± 0.15	5.1 ± 0.19	-1.5 (-1.83, -1.14)
Overall	19.0 ± 0.4	14.3 ± 0.57	-4.7 (-5.69, -3.62)

The majority of participants showed an improvement in score for the three summary measurements of pain, stiffness and physical functioning, and for the overall WOMAC score. Mean overall WOMAC score improved significantly (p < 0.001) from baseline to endpoint (Table 4). In addition, the change from baseline to endpoint in score for 'pain right now' (from the pain assessment questionnaire) showed a weak positive correlation with the change in overall WOMAC score (Spearman correlation coefficient: non-normalized 0.344 and normalized 0.384).

### Evaluation of safety

Adverse events occurring during the treatment phase and tapering off phase were those associated with strong opioid treatment (Table 5). Adverse events were reported by 6% (9/159) of patients during the run-in period, 65% (68/104) of patients during the treatment period and 25% (12/48) during the optional tapering off period. The study medication was permanently stopped in 25% (39) of cases, particularly because of nausea (53%), vomiting (47%) and dizziness (18%). (No falls or fractures were reported.) Withdrawal syndrome was reported in two cases (OA hip) during tapering off – one was mild, the other moderate, and both resolved without specific treatment. No deaths occurred. Two patients reported at least one serious adverse event during the treatment phase (severe asthenia and anorexia in one case, bronchitis in the other) and one experienced a serious adverse event during the tapering-off phase (hospitalization due to chest pain and arrythmia), but these were considered unrelated to the study drug. There were no clinically significant changes in vital signs during the study.

**Table 5:** Adverse events (AEs) reported during the treatment phase and tapering off phase by >5% of participants

**Preferred term**	**Total AEs n (%)**
Treatment phase (N = 104)	
Nausea	51 (32%)
Vomiting	41 (26%)
Somnolence	25 (16%)
Dizziness	14 (9%)
Constipation	10 (6%)
Asthenia	9 (6%)
Pruritus	8 (5%)
Tapering off phase (N = 42)	
Nausea	5 (10%)
Vomiting	3 (6%)

## Discussion

The study was intended to evaluate the utility of TDF under routine conditions and to investigate different practical issues, such as the usefulness of a test-period and use of concomitant antiemetic treatment during the first weeks. It was not designed as a primary efficacy study, since the efficacy of TDF in providing pain relief has already been demonstrated [14-18]. For this reason it was considered unethical to include a placebo control group.

A one-month test period [10] is sufficient to show a role for TDF in the treatment of pain caused by OA of the knee or hip that is not adequately controlled by NSAIDs, Cox-2 inhibitors, paracetamol or weak opioids at optimal doses. Findings support previous conclusions that OA-induced pain can be successfully treated with TDF, and that this may result in improved functioning [22]. Not all patients with OA of the knee or hip receive adequate doses of their current non-opioid analgesia because pain control can be improved in some when doses of these medications are optimized. Undertreatment of OA-related pain has been reported previously [24]. Adequate pain control was achieved for most patients after one week of TDF, at the starting dose of 25 μg/hr in over half of all patients, and relief was maintained over the treatment period. Pain was controlled in 88% of patients after one month with nearly 40% reporting mild or no pain.

A few patients started treatment with TDF, despite adequate pain control during the run-in phase. These patients were considered to be protocol violators. However, their inclusion provided the opportunity to determine whether TDF could give additional benefits to these patients above those already gained by their current medications. About half of these patients achieved further pain reduction while treated with TDF.

Over the treatment period, the numbers of all patients using other analgesics, especially NSAIDs decreased substantially (from 45% to 19%). This may be beneficial in reducing side effects such as gastrointestinal bleeding. Nearly all patients taking weak opioids took them on the first day of treatment only when serum levels of fentanyl had not yet achieved steady state. Paracetamol may be useful for breakthrough pain in some patients but need for rescue medication should be evaluated on an individual basis. Metoclopramide did not appear to prevent nausea or vomiting.

Treatment was considered favourable in terms of efficacy, side effects and convenience. A preference for treatment with TDF over sustained release morphine has previously been shown by patients with chronic non-cancer pain [16].

The general health measure, SF-36, indicated that control of pain significantly improved both physical and mental components of quality of life. The improvement in mental health may be related to improved functioning which permits greater social activity. In spite of similar pain control, patients with OA of the hip appeared to have slightly more difficulty with stiffness and general mobility than those with OA of the knee, such as getting in/out of the bath or bed, probably due to the location and function of these joints.

WOMAC is a reliable, valid and responsive multidimensional, self-administrated outcome measure designed specifically to evaluate patients with OA of the knee or hip [27]. Overall pain, stiffness and function significantly improved after one month of TDF treatment for both OA knee and OA hip patients, with improvements in nearly all items of WOMAC summary categories. Quality of life would be expected to be improved with pain relief, although significant pain relief would not necessarily be associated with reduced stiffness and increased physical function [29]. Increased functioning was also indicated by the fact that half of all patients required less help with daily activities of living. Return of a full range of motion is unlikely to occur when marked structural damage of the joint has occurred.

Overall, the spectrum of reported side effects was consistent with those commonly associated with opioid therapy and with previous experience with TDF. Constipation was not a problem for this patient population as with other patients receiving TDF for non-cancer pain or cancer pain [16,29,30], and tolerance to other side effects is likely to develop with continued treatment [31]. In addition to nausea and vomiting, patients should be warned of the possibility of dizziness when starting strong opioid treatment. This is especially important for the elderly OA population in order to prevent falls. In the present study, 9% of patients reported dizziness.

As might be expected in an elderly population, many patients had co-existing diseases. For example, 59% had cardiovascular disease on entry, which is of particular interest given the concern about the cardiovascular safety of some Cox-2 inhibitors which, since this trial was undertaken, has led to the withdrawal of rofecoxib.

This trial demonstrates that patients with OA of the knee or hip continue to experience pain even at optimal doses of their non-opioid treatment, providing a major reason why patients and clinicians alike are often dissatisfied with current therapies. The study also shows that patients with OA benefit from additional pain control provided by TDF [32]. Clinicians are beginning to accept that strong opioids are well tolerated and effective and should be made available when non-opioids have failed to control pain [33]. Our findings support this reasoning and suggest that opioids should be made more widely available where appropriate.

## Statement of competing interest

Dr Richarz is an employee of Janssen-Cilag and the other authors have both received research funding from Janssen-Cilag, which funded this study.

## Authors' contributions

UR designed the study and coordinated it. XLL and KP recruited patients to the study and contributed to the interpretation of the findings. All authors contributed to developing the manuscript for publication.

## Pre-publication history

The pre-publication history for this paper can be accessed here:


